# CCL5 Deficiency Enhanced Cryo–Thermal-Triggered Long-Term Anti-Tumor Immunity in 4T1 Murine Breast Cancer

**DOI:** 10.3390/biomedicines10030559

**Published:** 2022-02-26

**Authors:** Yue Lou, Shengguo Jia, Ping Liu, Lisa X. Xu

**Affiliations:** School of Biomedical Engineering and Med-X Research Institute, Shanghai Jiao Tong University, Shanghai 200030, China; louyue4@163.com (Y.L.); 18391861735@163.com (S.J.)

**Keywords:** CCL5, cryo–thermal therapy, breast cancer, anti-tumor immunity

## Abstract

Breast cancer remains one of the most common solid tumors. Tumor immunosuppressive factors mainly hinder the control of tumors. We previously developed an innovative cryo–thermal therapy that was shown to significantly suppress distal metastasis and improve long-term survival in murine B16F10 melanoma and 4T1 mammary carcinoma models. However, the effect of cryo–thermal therapy on the 4T1 model was not excellent. CCL5 has been reported to help the progression of breast cancer, so in this study, CCL5^−/−^ was used to explore the role of host-derived CCL5 after cryo–thermal therapy. CCL5^−/−^ could not completely resist tumor development, but it significantly improved survival rates when combined with cryo–thermal therapy. Mechanically, CCL5^−/−^ mildly decreases the percentage of MDSCs, increases DC maturation and macrophage’s inflammatory function at an early stage after tumor inoculation, and later up-regulate the level of Th1 and down-regulate the level of Tregs. When combined with cryo–thermal therapy, CCL5^−/−^ dramatically down-regulated the proportion of MDSCs and induced full M1 macrophage polarization, which further promoted Th1 differentiation and the cytotoxicity of CD8^+^ T cells. Our results indicated that CCL5^−/−^ contributed to cryo–thermal-triggered, long-lasting anti-tumor memory immunity. The combination of cryo–thermal therapy and CCL5 blockades might extend the survival rates of patients with aggressive breast cancer.

## 1. Introduction

Tumor immunosuppression is pivotal in immune escape and the initiation of metastasis [1,2]. A variety of immune-suppressive cells, including myeloid-derived suppressor cells (MDSCs), tumor-associated macrophages, regulatory dendritic cells and regulatory T cells (Tregs), are recruited to establish tumor immunosuppression [3,4,5]. Tumor-induced immunosuppression can impair antigen presentation, inactivate co-stimulatory signals, secrete immune-regulatory cytokines and induce tolerance [6], and it is a hindrance for the rejection of local tumor growth and distant metastasis [7]. Therefore, it is essential for effective cancer treatment to reverse tumor immunosuppression and sustain durable anti-tumor immunity.

Accumulating therapeutic interventions that target tumor immunosuppressive factors are considered to be effective therapeutic strategies for metastatic cancer [8]. The CCL5/RANTES chemokine is well-known as an important contributor for tumor invasion and metastasis [9]. Several studies have shown that a high level of CCL5 induces tumor cell proliferation, migration and angiogenesis [9,10,11]. In addition, CCL5 can mediate the recruitment and chemotaxis of Tregs [12], macrophages [13], DCs [14] and MDSCs [15], as well as induce the Th2 polarization of CD4^+^ T cells [16], which consequently promote tumor growth and metastasis. An elevated level of CCL5 in various cancer patients indicates a poor prognosis [17]. CCL5 represents an efficacious therapeutic target to prevent cancer recurrence and metastasis [8].

In our previous study, an innovative tumor therapeutic strategy named cryo–thermal therapy was established to ablate primary tumors through the rapid cooling and heating of local tumor tissue [18,19,20,21]. This treatment effectively inhibited distal lung metastasis and markedly prolonged the long-term survival in 4T1 mice mammary carcinoma and B16F10 melanoma models [22,23]. The therapy also resulted in a significant reduction in MDSCs, the activation and maturation of DCs, and the polarization of M1 macrophage, which are essential for CD4^+^ T cell-mediated, adaptive, and durable anti-tumor immunity [24]. However, the survival rate of the 4T1 model after cryo–thermal therapy was not excellent [22]. To completely reverse host immunosuppression and improve therapeutic efficacy in malignant invasive tumor, it is necessary to combine cryo–thermal therapy with another therapeutic intervention, such as small-molecule targeted drugs. Recently, CCL5 antagonists were shown to be useful in clinical cancer immunotherapy [25]. Therefore, to investigate the role of CCL5 in cryo–thermal-induced anti-tumor memory immunity, we explored the therapeutic effect of cryo–thermal therapy on CCL5^−/−^ mice with the 4T1 model. Our study showed that CCL5^−/−^ effectively improved the survival rate of cryo–thermal-treated mice and CCL5^−/−^ further reduced the level of MDSCs and promoted the phenotypic maturation of DCs, the polarization of M1 macrophage, the functional differentiation of CD4^+^ T cells, and the generation of cytotoxic CD8^+^ T cells. CCL5^−/−^-induced M1 macrophage polarization played a crucial role in the inhibition of Tregs and Th2 cell differentiation and the cytotoxic of CD8^+^ T cells, which would be exclusively responsible for mediating cryo–thermal-triggered, long-lasting anti-tumor memory immunity. Our study revealed that the targeted inhibition of CCL5 could be applied in combination with cryo–thermal therapy to improve therapeutic efficacy in malignant invasive breast cancer.

## 2. Materials and Methods

### 2.1. Cell Culture

4T1 cells were obtained from Shanghai First People’s Hospital, China and cultured in a DMEM medium (Hyclone, Logan, UT, USA) supplemented with 10% FBS, 100 U/mL of penicillin, and 100 μg/mL of streptomycin (Shanghai Sangon, Shanghai, China) at 37 °C in a 5% CO_2_ incubator.

### 2.2. Animal Model

The female BALB/c mice were purchased from Slaccas (Shanghai, China). CCL5^−/−^ transgenic mice were donated by Dr. Yan Zhang from Med-X Research Institute, Shanghai Jiao Tong University. The use of the CCL5^−/−^ transgenic mouse model has been published in Cancer Research [16] and Cell Death & Disease [26]. All animal experiments were approved by the Animal Welfare Committee of Shanghai Jiao Tong University (license No. 2020017), and all experimental methods were in accordance with the guidelines of Shanghai Jiao Tong University Animal Care and the ARRIVE guidelines. To prepare the tumor-bearing mice, 6–8-week-old mice were used, and 5 × 10^5^ 4T1 cells were injected s.c. into the right femoral region of each mouse.

### 2.3. The Procedures of Cryo–Thermal Therapy

The system of cryo–thermal therapy was developed by Dr. Aili Zhang and Engr. Jincheng Zou at Med-X Research Institute, Shanghai Jiao Tong University. On day 21 after inoculation (about 10 mm in diameter), mice were randomly divided into a tumor-bearing group (control) and a cryo–thermal group (cryo–thermal). The tumors of mice in the treated group were firstly frozen with LN_2_ and reached a temperature of −20 °C, and then they were heated by RF and maintained at 50 °C for 10 min.

### 2.4. Flow Cytometry (FACS) Analysis

Mice were sacrificed at the indicated time after treatment. Cells from the spleen and peripheral blood of mice were stained with fluorescence-conjugated antibodies. For intercellular staining, cells were stimulated for 4 h with a Cell Activation Cocktail (with Brefeldin A, San Diego, CA, USA). After surface staining, cells were fixed and permeabilized (BioLegend, San Diego, CA, USA) before being stained with antibodies. The data were collected with a BD FACS Aria II cytometer (BD Biosciences) and analyzed using FlowJo 7 software (https://www.flowjo.com, accessed on 10 February 2022). A list of used fluorochrome-conjugated monoclonal antibodies is shown in Table 1 and the gating strategy for immune cell subpopulations was shown in Figure S3.

### 2.5. RNA Isolation and Real-Time PCR

TRIzol Reagent (TaKaRa, Japan) was used for total RNA isolation. The absorbance at 260/280 nm of samples above 1.9 were used to obtain cDNA via the PrimeScript RT reagent kit (TaKaRa, Otsu, Shiga, Japan). SDS 2.4 software (Applied Biosystems, Waltham, MA, USA) was used for Ct value collection. The expression level of targeted genes was normalized as GAPDH (ΔΔCt method).

### 2.6. In Vitro Co-Culture Assay

On day 24 after inoculation, isolation kits were used for obtaining the CD4^+^ and CD8^+^ T cells from wild-type (WT) control mice. For macrophage isolation, splenocytes from WT control mice on day 24 after inoculation or the CCL5^−/−^ group (day 3 after therapy) were stained with the PE-CD68 monoclonal antibody and then isolated using a PE-positive selection kit. The T cells were co-cultured with macrophages at a ratio of 5:1, respectively, in 24-well plates for 24 h (5 × 10^5^ CD4^+^ or CD8^+^ T cells: 1 × 10^5^ macrophages per well in 1 mL of medium). For the proliferation assay, splenic CD4^+^ or CD8^+^ T cells were labelled with CFSE (5 μM/mL) and co-cultured with macrophages for 24 h. All the isolation kits were purchased from StemCell (Vancouver, BC, Canada).

### 2.7. Statistical Analysis

GraphPad Prism 9 (https://www.graphpad.com, accessed on 10 February 2022) was used for statistical analyses. Data for FACS were processed using one-way ANOVA, data for qRT-PCR were processed using two-way ANOVA, and the log-rank test was used to generate Kaplan–Meier survival curves; * *p* < 0.05, ** *p* < 0.01, and *** *p* < 0.001.

## 3. Results

### 3.1. CCL5 Deficiency Significantly Prolonged Long-Term Survival after Cryo–Thermal Therapy

**Figure 1:**
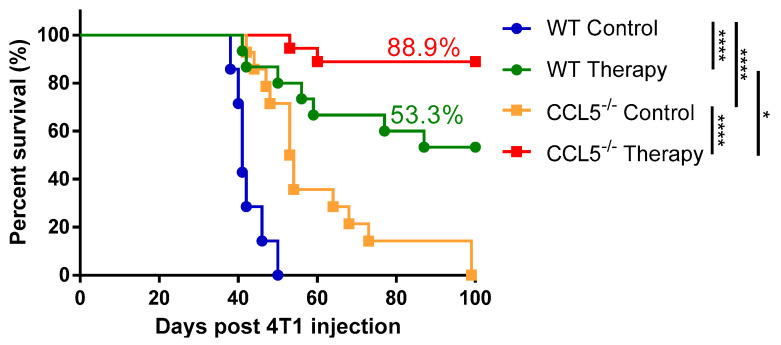
CCL5 deficiency prolonged the long-term survival of cryo–thermal therapy in the 4T1 model. Wild-type (WT) or CCL5 deficiency (CCL5^−/−^) mice were subcutaneously inoculated with 5 × 10^5^ 4T1 tumor cells and treated with cryo–thermal therapy on day 21 after inoculation. * *p* < 0.05; **** *p* < 0.0001. (*n* = 7 in WT control; *n* = 15 in WT therapy; *n* = 14 in CCL5^−/−^ control; *n* = 18 in CCL5^−/−^ therapy).

To understand the role of host-derived CCL5 in cryo–thermal therapy triggering anti-tumor immunity, the 4T1 breast cancer model was established in WT and CCL5^−/−^ transgenic mice and treated with cryo–thermal therapy. Untreated WT mice (WT control) mostly died within 50 days after inoculation (median survival time: 41 days) and the median survival time of tumor-bearing CCL5^−/−^ mice (CCL5^−/−^ control) was 54 days, but all mice died because of tumor burden. However, the survival rates were significantly improved in both WT and CCL5^−/−^ mice when treated with cryo–thermal therapy. Moreover, in treated groups, CCL5^−/−^ significantly improved the survival rate in comparison to WT mice (Figure 1). These results indicated that CCL5^−/−^ drastically improved the curative effects and prolonged the survival of cryo–thermal treated mice.

### 3.2. CCL5 Deficiency Enhanced DC Activation at an Early Stage after Cryo–Thermal Therapy

CCL5 participates in recruitment of immature DCs [27], which can induce T cell anergy and promote the differentiation of Tregs [28]. In this regard, we examined the activation and immune-stimulatory signature of DCs. Because the primary tumors after treatment were entirely ablated, tumor samples could not be collected, so we only focused on studying the systemic effects of CCL5 after treatment in the spleen and blood. On day 3 after treatment, the percentage of splenic DCs in the WT mice was significantly down-regulated, but it up-regulated on day 21. However, in CCL5^−/−^ mice, no obvious change was seen on day 3 after treatment compared to CCL5^−/−^ control mice (Supplementary Figure S1B). Similarly, the percentage of DCs in peripheral blood was down-regulated in WT mice but increased on day 5 in CCL5^−/−^ mice after treatment (Supplementary Figure S1C) compared to the CCL5^−/−^ control mice. These results suggested that CCL5^−/−^ could increase the proportion of DCs in the peripheral blood and spleen at an early stage after cryo–thermal therapy.

**Figure 2:**
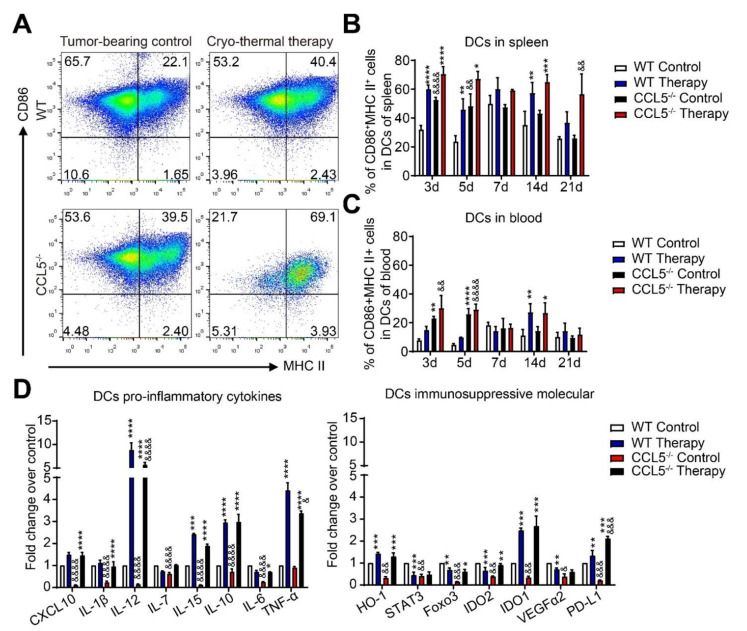
CCL5 deficiency enhanced the phenotypic maturation of DCs at an early stage after therapy. (**A**) Gating strategy of mature DCs. The percentages of mature DCs in the spleen (**B**) and blood (**C**) were evaluated at the indicated time points (3, 5, 7 and 21 d after treatment). (**D**) The expression of the immune-response-related molecules of DCs were detected with qRT-PCR. Total RNAs were isolated from splenic CD11c^+^ DCs in treated mice or control mice. *n* = 4 per group; mean ± SD. The data of FACS were analyzed with FlowJo 7. * *p* < 0.05; ** *p* < 0.01; *** *p* < 0.001; **** *p* < 0.0001, compared to WT tumor-bearing control or CCL5^−/−^ tumor-bearing control. & *p* < 0.05; && *p* < 0.01; &&& *p* < 0.001; &&&& *p* < 0.0001, compared to WT group.

Despite the reduction in DCs in WT mice (Supplementary Figure S1), the proportion of matured DCs (CD11c^+^CD86^+^MHC-II^+^) in the spleen and blood in both WT and CCL5^−/−^ mice was increased after treatment, but the level in treated CCL5^−/−^ mice was significantly higher than that of the treated WT mice in blood on days 3 and 5 (Figure 2B,C). The gating strategy is shown in Figure 2A. In particular, CCL5^−/−^ could maintain the high level of mature DCs in the spleens of mice after treatment (Figure 2B,C). These results suggested that CCL5 deficiency enhanced the cryo–thermal-induced phenotypic maturation of DCs.

We further evaluated the immune-stimulatory signature of splenic DCs. In the CCL5^−/−^ control mice, the expression of pro-inflammatory molecules (CXCL10, IL-1β, IL-12, IL-7, IL-15, IL-10 and IL-6) and immunosuppressive molecules (HO-1, STAT3, Foxo3, IDO2, IDO1, VEGFR2 and PD-L1) was significantly down-regulated compared to that in WT control mice. However, there were no significant differences between CCL5^−/−^ and WT treated mice, except for the lower levels of IL-12 and TNF-α and the higher level of PD-L1 in CCL5^−/−^ treated mice compared to the WT treated mice (Figure 2D). These results suggested that CCL5^−/−^ mainly enhanced cryo–thermal-induced DCs phenotypic maturation, not the function of mature DCs.

### 3.3. CCL5 Deficiency Enhanced the Cryo–Thermal-Induced Polarization of M1 Macrophages

CCL5 can increase the expression of IL-10 in macrophages [29], which is the hallmark of M2 macrophages [30]. Thus, we also evaluated the effect of CCL5 deficiency on macrophages after treatment. Likewise, compared to WT, a decreased percentage of macrophage in the spleen was found on day 3 in the CCL5^−/−^ control mice (Supplementary Figure S2).

**Figure 3:**
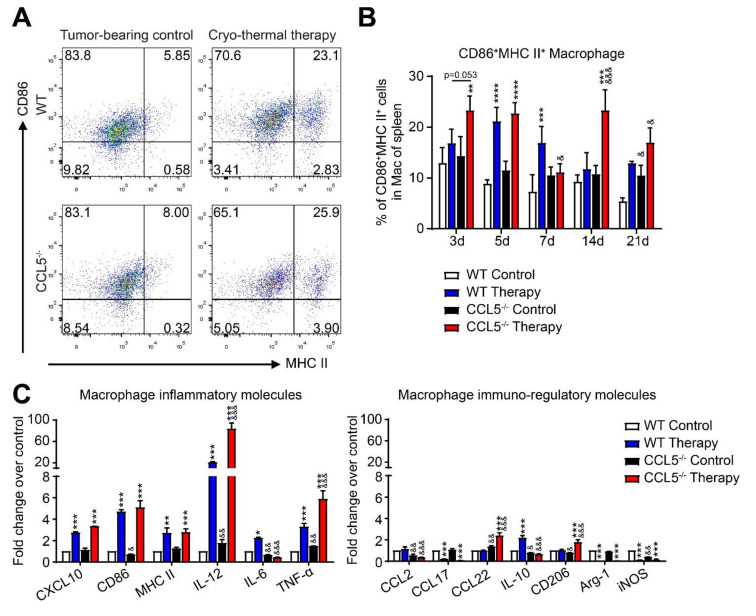
CCL5 deficiency promoted M1 macrophage polarization at an early stage after therapy. (**A**,**B**) FACS gating strategy (**A**) and analysis of the percentage of M1 macrophages (**B**) in the spleen were performed at indicated time points (3, 5, 7, 14 and 21 after therapy). (**C**) The expression of inflammatory and immune-regulatory molecules of macrophages were detected with qRT-PCR. Total RNAs were isolated from splenic CD68^+^ macrophages in treated mice or tumor-bearing mice. *n* = 4 per group; mean ± SD. The data of FACS were analyzed with FlowJo 7. * *p* < 0.05; ** *p* < 0.01; *** *p* < 0.001; **** *p* < 0.0001, compared to WT tumor-bearing control or CCL5^−/−^ tumor-bearing control. & *p* < 0.05; && *p* < 0.01; &&& *p* < 0.001, compared to WT group.

The proportions of polarized M1 macrophages (CD11b^+^F4/80^+^CD86^+^MHC-II^+^) in the spleen after treatment were significantly increased in both WT (on days 5 and 7) and CCL5^−/−^ mice (on days 3, 5 and 14). Importantly, CCL5^−/−^ enhanced the cryo–thermal-triggered M1 macrophage polarization on days 3 and 14 after therapy (Figure 3A,B), which implied that the M1 macrophage polarization in CCL5^−/−^ mice occurred much earlier and was stronger than that in WT mice after therapy.

Compared to the WT control mice, higher levels of M1-related cytokines (IL-12 and TNF-α) and lower levels of inflammatory molecules (CD86 and IL-6) and immune-regulatory molecules (CCL2, IL-10, CD206 and iNOS) were detected in CCL5^−/−^ control mice (Figure 3B,C). After treatment, the expression of IL-12, TNF-α, CCL22 and CD206 was up-regulated and the expression of IL-6, CCL2 and IL-10 was down-regulated in the CCL5^−/−^ mice compared to the WT treated mice (Figure 3C). These results indicated that CCL5^−/−^ may effectively promote M1 macrophage polarization at an early stage after treatment. Importantly, the effect of CCL5 deficiency on M1 polarization was much stronger than that on DC maturation in the spleen after treatment.

### 3.4. CCL5 Deficiency Induced the Reduction in Immunosuppressive MDSCs after Cryo–Thermal Therapy

**Figure 4:**
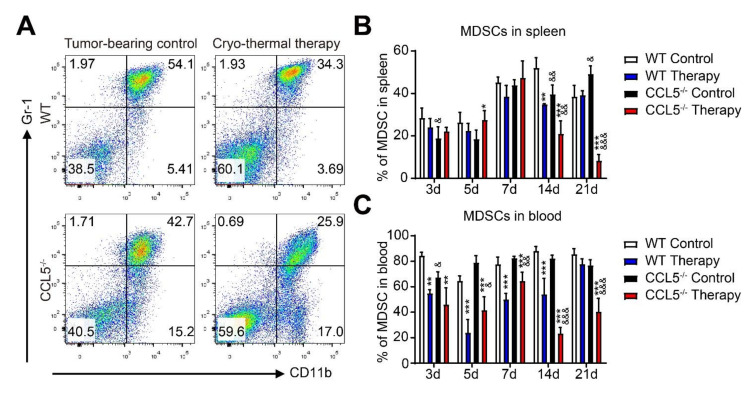
CCL5 deficiency down-regulated the proportion of MDSCs after treatment. (**A**) FACS strategy for the determination of the CD11b^+^Gr-1^+^ MDSC proportion. Flow-cytometry analysis of the MDSC proportion in the spleen (**B**) and blood (**C**) was performed at indicated time points. *n* = 4 per group; mean ± SD. The data of FACS were analyzed with FlowJo 7. * *p* < 0.05; ** *p* < 0.01; *** *p* < 0.001, compared to WT tumor-bearing control or CCL5^−/−^ tumor-bearing control. & *p* < 0.05; && *p* < 0.01; &&& *p* < 0.001, compared to WT group.

MDSCs drive cancer escape by inhibiting the activity of T cell adaptive immunity [31], while CCL5 is crucial for the generation and growth of MDSCs [8,17]. The gating strategy is shown in Figure 4A. Compared to the WT control mice, the level of MDSCs in the spleen and blood was mildly down-regulated on day 3 of treatment. After treatment, the percentage of MDSCs was down-regulated in the blood from days 3 to 14 and in the spleen on days 14 and 21 in both the WT and CCL5^−/−^ mice. Moreover, the proportion of MDSCs in the CCL5^−/−^ mice was much lower than that in the WT group (days 14 and 21 in the spleen and blood; Figure 4B,C). These results suggested that CCL5^−/−^ markedly down-regulated the level of MDSCs in both the spleen and blood, as well as perfectly reversing tumor immunosuppression after treatment.

### 3.5. CCL5 Deficiency Modulated the Poly-Functional Differentiation of CD4^+^ T Cells after Cryo–Thermal Therapy

**Figure 5:**
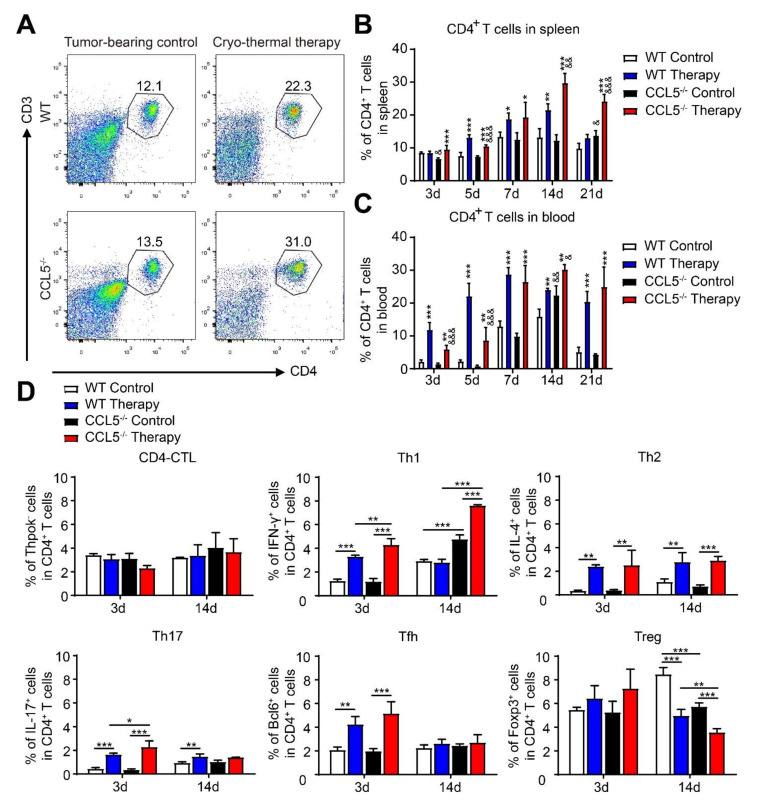
CCL5 deficiency strengthened cryo–thermal-induced the Th1 differentiation and inhibited Treg differentiation of CD4^+^ T cells. (**A**) Gating strategy for CD3^+^CD4^+^ cells. Analysis of the CD4^+^ T cell proportion in the spleen (**B**) and blood (**C**) was performed at indicated time points (3, 5, 7, 14 and 21 d after therapy). (**D**) The percentages of CD4^+^ T cell subsets after therapy were detected with FACS. *n* = 4 per, mean ± SD. The data of FACS were analyzed with FlowJo 7. * *p* < 0.05; ** *p* < 0.01; *** *p* < 0.001, compared to WT tumor-bearing control or CCL5^−/−^ tumor-bearing control. & *p* < 0.05; && *p* < 0.01; &&& *p* < 0.001, compared to WT group.

In addition to influencing innate cells, CCL5 promotes tumor metastasis via the induction of CD4^+^ T cell polarization [16] and enhances the infiltration and function of Tregs [32]. Thus, the percentages of CD4^+^ T cells in the spleen and blood after treatment were analyzed. The gating strategy is shown in Figure 5A. Similar levels of CD4^+^ T cells were found in both the spleen and blood from the WT and CCL5^−/−^ tumor-bearing mice (Figure 5B,C). After treatment, the amount of splenic CD4^+^ T cells in the WT mice was significantly increased on days 5, 7 and 14, but there was a downward trend on day 21. However, in the CCL5^−/−^ mice, the amount increased at every time point after therapy and reached a higher level than that in the WT treated mice (Figure 5B). In the peripheral blood, CD4^+^ T cells in both the WT and CCL5^−/−^ mice was increased at every time point after treatment but was also declined from days 14 to 21 in the WT treated mice; meanwhile a high level of CD4^+^ T cells was maintained in the CCL5^−/−^ treated mice (Figure 5C).

The plasticity of CD4^+^ T cells has been reported to response the environmental cues [33]. Therefore, we tested the change of splenic CD4^+^ T cell lineages after treatment. The CD4^+^ T cell subsets showed no difference between the WT and CCL5^−/−^ tumor-bearing mice on day 3, but on day 14, the CCL5^−/−^ tumor-bearing mice had higher levels of Th1 and lower levels of Tregs compared to the WT control mice. After treatment, the percentages of Th1, Tfh, Th17 and Th2 in both the WT and CCL5^−/−^ mice were increased on day 3. Meanwhile, the CCL5^−/−^ mice showed higher proportions of Th1 and Th17 compared to the WT group (Figure 5D). On day 14 after treatment, Th1% in the spleens of the WT mice was decreased and had no difference compared to the control mice, and a high level of Th1 lineage was still maintained in the CCL5^−/−^ mice. Moreover, a significantly lower proportion of Tregs was observed in the CCL5^−/−^ mice compared to the WT mice after treatment (Figure 5D). These results indicated that CCL5^−/−^ is beneficial to better CD4^+^ T cell responses in long-term tumor stimulation and could strengthen the Th1-dominant differentiation of CD4^+^ T cells.

### 3.6. CCL5 Deficiency Promoted the Generation of Cryo–Thermal Therapy-Induced Cytotoxic CD8^+^ T Cells

**Figure 6:**
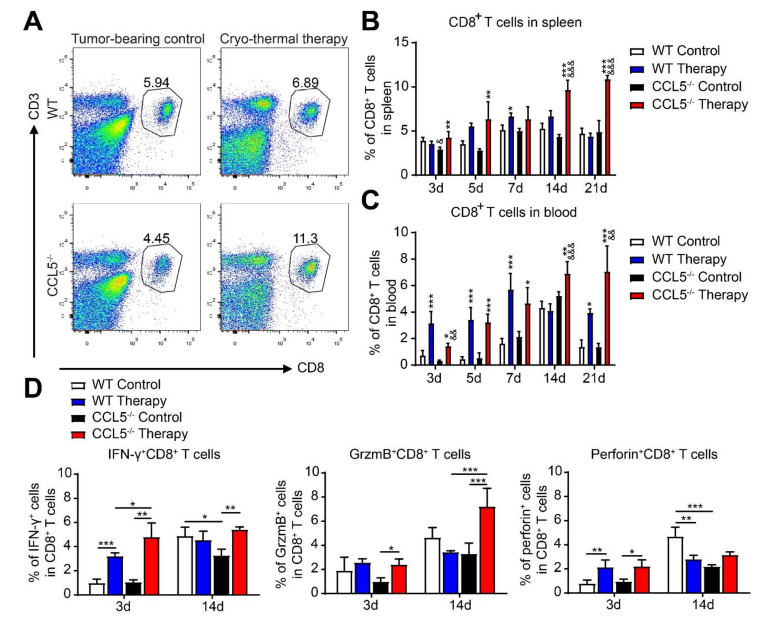
CCL5 deficiency promoted the cryo–thermal therapy-induced cytotoxic CD8^+^ T cells after treatment. (**A**) Gating strategy for the determination of CD3^+^CD8^+^ T cells in the spleen and blood. Analysis of the CD8^+^ T cell proportions in the spleen (**B**) and peripheral blood (**C**) after treatment. (**D**) The expression levels of the cytotoxic cytokines of CD8^+^ T cells on days 3 and 14 after treatment were detected with FACS. *n* = 4 per group; mean ± SD. The data of FACS were analyzed with FlowJo 7. * *p* < 0.05; ** *p* < 0.01; *** *p* < 0.001, compared to WT tumor-bearing control or CCL5^−/−^ tumor-bearing control. & *p* < 0.05; && *p* < 0.01; &&& *p* < 0.001, compared to WT group.

We next investigated the change of CD8^+^ T cells in the CCL5^−/−^ mice after therapy. The gating strategy is shown in Figure 6A. Similar levels of CD8^+^ T cells ere found in both the spleen and blood of the WT and CCL5^−/−^ tumor-bearing mice (Figure 6B,C). After treatment, we found increasing and decreasing trends of splenic CD8^+^ T cells on days 7 and 21, respectively, in the WT mice; however, in the CCL5^−/−^ group, the percentage of splenic CD8^+^ T cells persistently increased before day 14 and maintained a high level on day 21 (Figure 6B). In peripheral blood, cryo–thermal therapy increased the percentage of CD8^+^ T cells on days 3, 5, 7 and 21 in the WT mice and at every time point in CCL5^−/−^ mice. Similar to the change of CD4^+^ T cells, the percentage of CD8^+^ T cells declined in the WT mice, while a higher level of CD8^+^ T cells in CCL5^−/−^ mice was observed from days 14 to 21 after treatment (Figure 6C).

Next, we analyzed the change of representative cytotoxic factors in CD8^+^ T cells. The splenic CD8^+^ T cells of the WT and CCL5^−/−^ control mice presented similar expressions of IFN-γ, Granzyme-Bm and perforin on day 3, but on day 14, the expression of IFN-γ and perforin was decreased in the CCL5^−/−^ control mice compared to the WT control mice (Figure 6D). On day 3 after treatment, the levels of IFN-γ and perforin in WT mice increased compared to those of the WT control, while in the CCL5^−/−^ mice, the cryo–thermal therapy further up-regulated Granzyme-B (Figure 6D). In addition, a higher level of IFN-γ was found in the CCL5^−/−^ treated mice in comparison to the WT treated mice. On day 14 after therapy, the level of perforin was down-regulated in the WT mice and the levels of IFN-γ and Granzyme B in the CCL5^−/−^ treated mice maintained higher levels than that in the CCL5^−/−^ control. Specifically, CCL5^−/−^ resulted in a higher Granzyme-B levels than those in WT treated mice on day 14 (Figure 6D). These results indicated that CCL5^−/−^ could further promote the cytotoxic of CD8^+^ T cells.

### 3.7. CCL5-Deficiency-Enhancing, Cryo–Thermal-Induced M1 Macrophage Was Required for Inhibiting the Differentiation of CD4^+^ T Cell into Suppressive Subsets In Vitro

**Figure 7:**
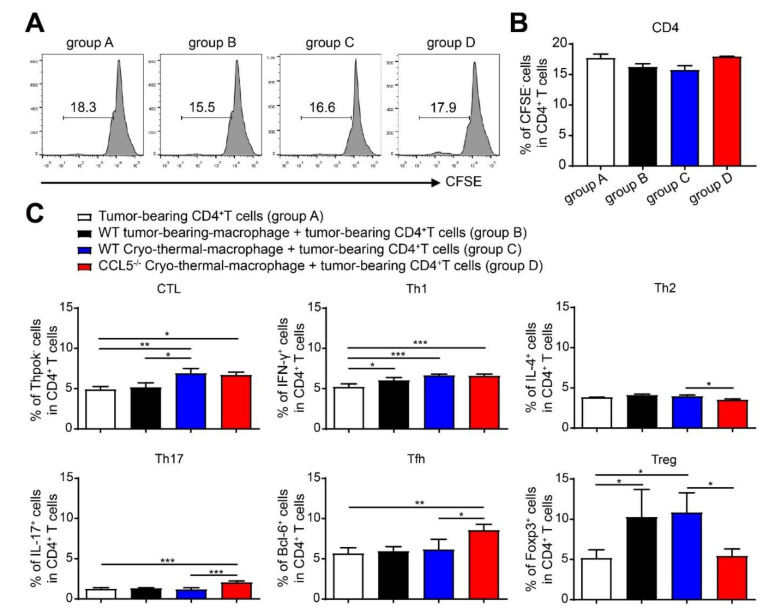
CCL5-deficiency-enhanced M1 polarization was required to inhibit CD4^+^ T cell differentiation into Th2 and Tregs in vitro. Splenic CD4^+^ T cells from WT control mice were pre-treated with CFSE and then co-cultured with macrophages from the WT control, treated WT, or CCL5^−/−^ mice in vitro for 24 h. The gating strategy (**A**), CFSE^−^CD4^+^ T cells (**B**), and the proportion different CD4^+^ T cell subsets (**C**) were analyzed with FACS. *n* = 4; mean ± SD. The data were analyzed with FlowJo 7 and processed using one-way ANOVA in GraphPad prism 9, * *p* < 0.05; ** *p* < 0.01; *** *p* < 0.001.

The generation of systemic anti-tumor immune response depends on effective innate immune activation [34]. Our results suggested that the effect of CCL5^−/−^ on the cryo–thermal-induced polarization of M1 macrophages was much stronger than that of DCs. Thus, we hypothesized that the CCL5-deficiency-enhanced M1 polarization at an early stage was responsible for the subsequent activation of T cells. To test splenic macrophages isolated from the WT control mice, WT or CCL5^−/−^ treated mice were co-cultured with CD4^+^ T cells (isolated from WT control mice at day 24 after inoculation) termed groups B, C, and D. The control CD4^+^ T cells alone were considered the control (termed group A). No difference were observed in the proliferation of CD4^+^ T cells among all groups (Figure 7A,B). We then sought to investigate whether the polarization of M1 macrophages from treated CCL5^−/−^ mice could directly affect the plasticity of CD4^+^ T cells in vitro. The proportions of Tregs and Th2 in group D were lower than those in group C, while the Tfh and Th17 levels were higher in group D than those in group C. There was no significant difference in the proportions of CD4-CTL and Th1 between groups C and D, but both proportions were higher than that of group A (Figure 7C). These results indicated that, in addition to CD4^+^ T cell differentiation into CTL and Th1 sub-lineages, CCL5-deficiency-enhancing, cryo–thermal-induced M1 macrophage polarization strongly promoted the differentiation of CD4^+^ T cells into Tfh and Th17 sub-lineages and inhibited the differentiation into Tregs and Th2.

### 3.8. CCL5-Deficiency-Enhancing, Cryo–Thermal-Induced M1 Macrophage Polarization Promoted the Proliferation of CD8^+^ T Cells and Enhanced the Cytotoxicity of CD8^+^ T Cells In Vitro

**Figure 8:**
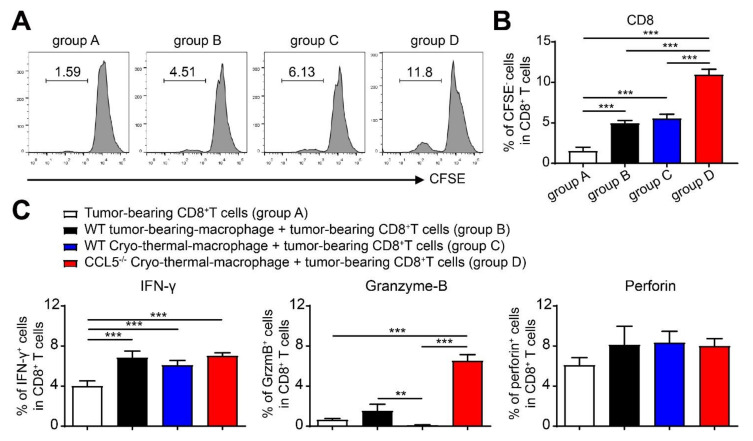
CCL5-deficiency-enhanced M1 polarization was required for the proliferation and cytotoxicity of CD8^+^ T cells. Splenic CD8^+^ T cells from WT control mice were pre-treated with CFSE and then cultured with macrophages from WT control mice, treated WT, or CCL5^−/−^ mice in vitro for 24 h. The gating strategy (**A**), percentage of CFSE^−^CD8^+^ T cells (**B**), and the expression level of IFN-γ, Granzyme B and perforin (**C**) were tested with FACS. n = 4; mean ± SD. The data of FACS were analyzed with FlowJo 7. Data were processed using one-way ANOVA in GraphPad Prism 9. ** *p* < 0.01; *** *p* < 0.001.

The same assay was performed to investigate CCL5^−/−^-enhancing M1 polarization in regulating the cytotoxicity of CD8^+^ T cells. The proliferation of CD8^+^ T cells was markedly induced when co-cultured with macrophages from WT treated mice, and it was especially prominent when co-cultured with the macrophages of CCL5^−/−^ treated mice (Figure 8A,B). Macrophages from WT treated mice or CCL5^−/−^ treated mice could enhance the expression of IFN-γ; specifically, higher level of IFN-γ and Granzyme-B could be induced when co-cultured with CCL5^−/−^ treated mice macrophages (Figure 8C). These results indicated that cryo–thermal therapy combined with CCL5^−/−^ could more effectively induce the polarization of M1 macrophage, which markedly promoted the proliferation and cytotoxicity of CD8^+^ T cells.

## 4. Discussion

In this study, we focused on the roles of CCL5^−/−^ in cryo–thermal-induced anti-tumor immunity against 4T1 mice breast cancer. Our results showed that CCL5^−/−^ could markedly improve the survival rate of treated mice. Mechanically, cryo–thermal therapy combined with CCL5 deficiency strongly triggered DC maturation and M1 macrophage polarization, markedly decreased the proportion of MDSCs, distinctly induced the Th1-dominant CD4^+^ T cell differentiation, and down-regulated CD4^+^ T cell differentiation into Tregs. Notably, the cryo–thermal-induced polarization of M1 macrophages in CCL5^−/−^ mice was required to strongly inhibit CD4^+^ T cell differentiation into Tregs and Th2 and to enhance the cytotoxicity of CD8^+^ T cells. These results suggested that targeting CCL5 in breast cancer could magnify the anti-tumor immunity triggered by cryo–thermal therapy.

The mouse 4T1 cell line is known for its low immunogenic, high tumorigenic, and invasive activities [35]. 4T1 can induce the generation of immunosuppressive environments [36]. Compared to other triple-negative breast cancer cell lines (such asEMT6 and Py230), 4T1 is more aggressive for primary tumors, and metastasis can be formed earlier [37,38]. To evaluate the therapeutic effect of cryo–thermal therapy in controlling tumor metastasis, the 4T1 model was used in this study. In our previous study, cryo–thermal therapy was found to activate the long-term systematic anti-tumor immune response, which led to a survival rate of over 80% in the B16F10 model [23]. However, this high survival rate could not be observed in the 4T1 model [19,22]. We suggested that a better therapeutic effect could not achieved in the 4T1 model after therapy probably because of the aggressive nature of 4T1, attributed to potent immunosuppression. We considered whether the therapeutic effect of cryo–thermal therapy could be improved in the 4T1 model with the addition of other agents used to alleviate the immunosuppression.

CCL5 expression is speculated to be part of an ongoing malignant process, but its function has not been fully described yet. CCL5 can increase the accumulation of MDSCs in tumors and improve their immunosuppressive function [39]. Moreover, CCL5 can induce the immunosuppressive polarization of macrophages [40], initiate the Th2 differentiation of CD4^+^ T cells [16] and recruit Tregs [12] to form tumor suppressive environments. These studies indicate that CCL5 represents a therapeutic target of breast cancer [41]. However, tumor-derived CCL5 does not act as the significant contributor to mammary carcinoma growth [42], and hematopoietic-derived CCL5 promotes tumor progression via the generation of MDSCs and maintenance of its immunosuppressive properties [41]. Thus, in this study, we focused on the host-derived CCL5 in immune response after treatment.

Considering the function of host-derived CCL5 in breast cancer progression and tumor immunosuppression, in the present study, CCL5-deficient mice (CCL5^−/−^) were used to establish the 4T1 model. CCL5^−/−^ was found to be able to increase the level of phenotypic mature DCs and promote the inflammatory function of macrophages at an early stage, as well as to mildly decrease the percentage of MDSCs, up-regulate the level of Th1, and down-regulate the level of Tregs at a late stage after tumor inoculation. However, CCL5^−/−^ only prolonged the survival time of 4T1-bearing mice. All the tumor-bearing mice in WT and CCL5^−/−^ mice died. These results indicated that CCL5^−/−^ could ameliorate tumor immunosuppression to a certain extent and delay tumor progression in comparison to WT mice, but it was not able to protect mice from malignant tumor deaths. However, cryo–thermal therapy was able to significantly improve the survival rate in CCL5^−/−^ mice, which suggested that the combination of cryo–thermal therapy and targeted inhibitors of CCL5 can further improve the survival rate of breast cancer therapy.

In this study, compared to WT mice, the percentage of MDSCs was much more decreased and the proportions of mature DCs, M1 macrophages, and CD4^+^ and CD8^+^ T cells were significantly increased in CCL5^−/−^ mice after treatment. Cryo–thermal therapy much further stimulated M1 macrophage polarization, promoted the differentiation of the Th1 and Th17 subsets, and enhanced the cytotoxicity of CD8^+^ T cells. Our study suggests that cryo–thermal therapy combined with targeting CCL5 can desirably reverse immunosuppression and elicit stronger host anti-tumor immunity, leading to improved cryo–thermal therapeutic efficacy against poorly immunogenic breast cancer.

In our previous study, repolarized M1 macrophages transformed the immunosuppressive into an acute inflammatory environment [43], facilitated the functional maturation of DCs, promoted Th1 differentiation, and increased the cytotoxicity of CD8^+^ T cells [24]. In this study, the effect of CCL5^−/−^ on M1 polarization was much stronger than that on DC maturation after treatment. CCL5^−/−^ further enriched the amount of M1 macrophages, which led to the differentiation of Th1 subsets, inhibited the differentiation of Tregs, and increased the cytotoxicity of CD8^+^ T cells. These results suggested that CCL5^−/−^-amplified, cryo–thermal-induced antitumor immune response could effectively contribute to M1 macrophage polarization. Whether cryo–thermal therapy combined with CCL5-deficiency-induced M1 macrophage polarization could contribute to decreasing the amounts of MDSCs and mature DCs should be further investigated.

In this study, the expression of CD206 and CCL22 was increased in CCL5^−/−^ mice after cryo–thermal therapy. CD206 is critical for complex glycan structure recognition and has evolved to facilitate antigen endocytosis and presentation [44]. The up-regulation of CD206 may promote tumor-related antigen uptake and the presentation of macrophages. Meanwhile, the high expression of CCL22 was found to promote the migratory activity of CD4^+^ T cells and enhance IFN-γ production by T cells [45]. The neutralization of CCL22 stimulated M2 macrophage polarization [46]. Hence, we suggested that the up-regulated expression of CCL22 and CD206 in CCL5^−/−^ mice after cryo–thermal therapy could play a positive role in anti-tumor activity of macrophages.

In this study, we mainly studied the role of host-derived CCL5 in the immune response after cryo–thermal therapy, and we are carrying out another study on combinations of cryo–thermal therapy and the antibody inhibition of CCL5/RANTES or CCR5 blockade in the 4T1 tumor model.

## 5. Conclusions

In summary, our results revealed that CCL5^−/−^ contributed to decreases in the proportion of MDSCs; promoted M1 macrophage polarization, which induced CD4^+^ T cell differentiation into Th1 subsets and cytotoxic CD8^+^ T cells when treated with cryo-thermal therapy; and proved the survival rate of 4T1-challenged mice. Our results suggested that the combined targeted inhibition of CCL5 could amplify the anti-tumor immunity induced by cryo–thermal therapy and have strong therapeutic impacts on 4T1 murine breast cancer. The biochemical aspects will be the subject of further studies.

## Figures and Tables

**Table 1:** Antibodies used in the FACS analysis.

Fluorescence Labeling	Antibodies	Clone
FITC	CD3	145-2C11
	CD11b	M1/70
	CD11c	N418
PE	Gr-1	R86-8C5
	IL-17	TC11-18H10.1
	Perforin	S16001B
	Foxp3	MF-14
	CD86 ^2^	GL-1
PerCP/Cy5.5	I-A/I-E	M5/114.15.2
	Bcl-6	7D1
PE/Cy7	CD25	3C7
APC	F4/80	BM8
AF647	ThPok ^1^	T43-94
APC/Cy7	CD4	RM4.5
	CD8	53-5.8
BV421	IL-4	11B11
BV510	IFN-γ	XMG1.2

^1^ The antibody was purchased from BD Biosciences. ^2^ The antibody was purchased from Sungene Biotech. Other reagents were purchased from BioLegend (San Diego, CA, USA).

## Data Availability

Data are contained within the article or supplementary material or are available from the authors upon reasonable request.

## Supplementary Materials

The following supporting information can be downloaded at https://www.mdpi.com/article/10.3390/biomedicines10030559/s1: Figure S1: CCL5 KO up-regulated the proportion of DCs at an early stage after therapy; Figure S2: CCL5 KO down-regulated the proportion of macrophages in the spleen on day 24 after tumor inoculation in tumor-bearing, CCL5-deficiency mice; Figure S3: Gating strategy for immune cell subpopulations.
