# Supplementary material for: CCL5 Deficiency Enhanced Cryo–Thermal-Triggered Long-Term Anti-Tumor Immunity in 4T1 Murine Breast Cancer
Source: Biomedicines. 2022 Feb 26;10(3):559. doi: 10.3390/biomedicines10030559 (PMC8945488; doi:10.3390/biomedicines10030559)
Supplement: Supplementary file 1 [file biomedicines-10-00559-s001.zip › biomedicines-1487182-supplementary.pdf]

Article

Supplementary Materials

CCL5 deficiency enhanced cryo-thermal-triggered long-term anti-tumor immunity in 4T1 murine breast cancer

Yue Lou [1], Shengguo Jia [1], Ping Liu [1,*] and Lisa X. Xu [1,*]

[1] School of Biomedical Engineering and Med-X Research Institute, Shanghai Jiao Tong University, Shanghai, China
* Correspondence: LXX: lisaxu@sjtu.edu.cn; PL: pingliu@sjtu.edu.cn

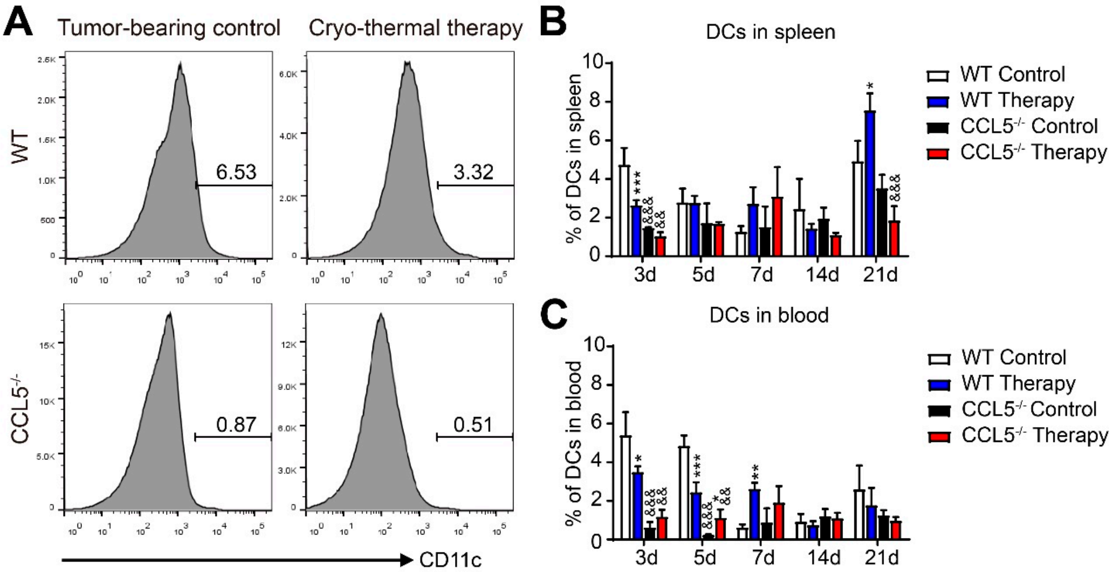

Supplementary Figure S1 CCL5 KO was up-regulated the proportion of DCs at early stage after therapy. (A) Flow cytometry gating strategy for determination of CD11c^{+}DCs in spleen and the peripheral blood. Flow-cytometry analysis of the dynamic change of DC proportion in spleen (B) and the peripheral blood (C) was performed at different time points (3d, 5d, 7d, 21d after cryo-thermal therapy) as compared to the tumor-bearing control group. n = 4 mice at each time point per group. Data was shown as mean ± SD. *p < 0.05; **p < 0.01; ***p < 0.001 was considered to be statistically significant as compared to WT tumor-bearing control. $^{\&}$p < 0.05; $^{\&\&}$p < 0.01; $^{\&\&\&}$p < 0.001 was considered to be statistically significant as compared to cryo-thermal treated WT or cryo-thermal treated CCL5$^{-/-}$ mice.

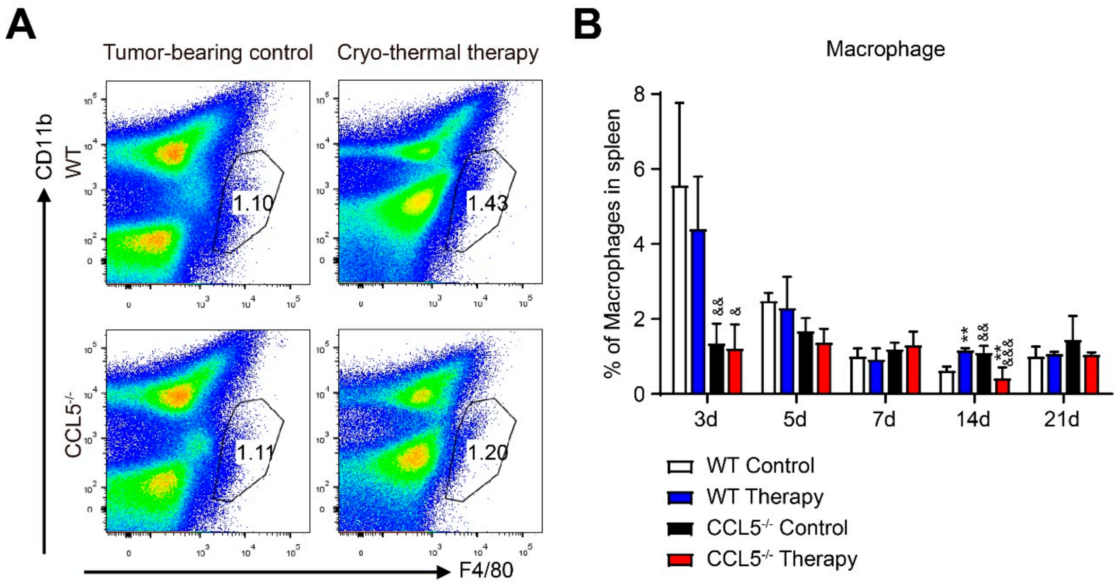

Supplementary Figure S2 CCL5 KO down-regulated the proportion of macrophages in spleen on day 24 after tumor inoculation in tumor-bearing CCL5 deficiency mice. (A) Flow cytometry gating strategy for determination of the $CD11b^+F4/80^+$ macrophage proportion in spleen. (B) Flow-cytometry analysis of the percentages of macrophages in spleen was performed at different time points (3d, 5d, 7d, 14d, 21d after cryo-thermal therapy) as compared to the tumor-bearing control group. n = 4 mice at each time point per group. Data was shown as mean ± SD. $^*p < 0.05$; $^{**}p < 0.01$; $^{***}p < 0.001$ was considered to be statistically significant compared to WT tumor-bearing control. $^{\&}p < 0.05$; $^{\&\&}p < 0.01$; $^{\&\&\&}p < 0.001$ was considered to be statistically significant compared to WT cryo-thermal treated or $CCL5^{-/-}$ cryo-thermal treated group.

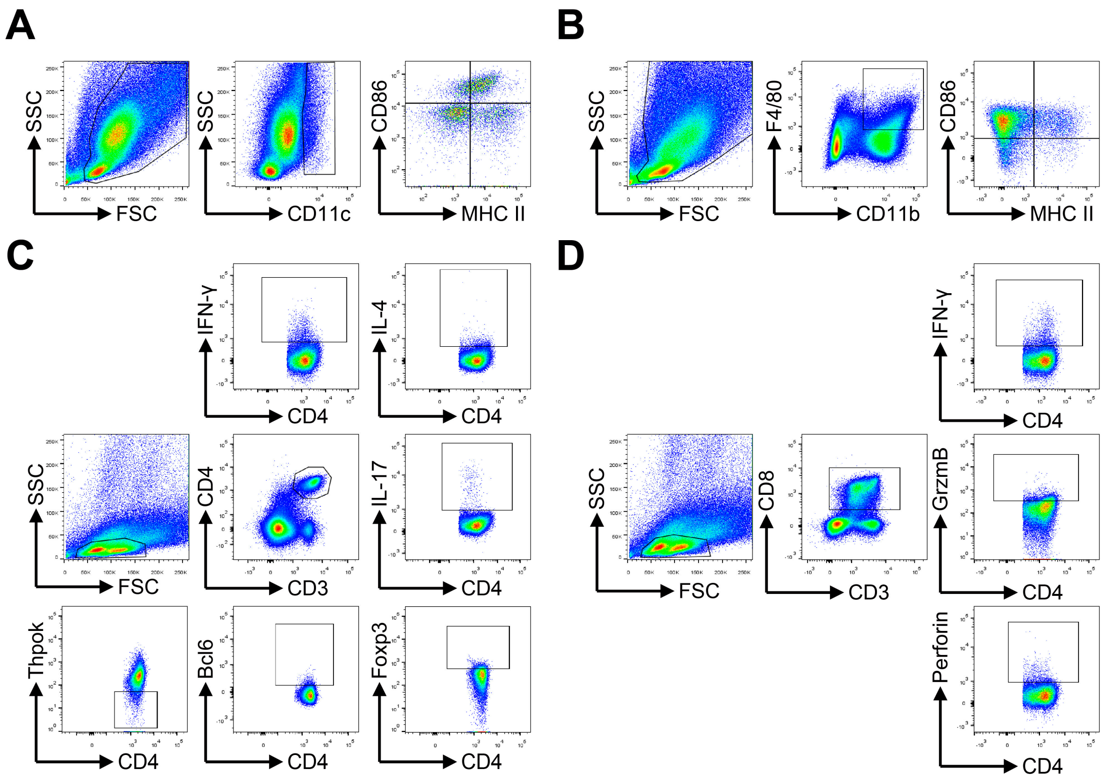

Supplementary Figure S3 Gating strategy for immune cell subpopulations. Flow cytometric gating strategy to identify DCs (A), macrophages (B), CD4 subsets (C) and CD8 subsets (D) through surface marker, cytokine and transcriptional factor expression.
